# Growth and Competitive Effects of *Centaurea stoebe* Populations in Response to Simulated Nitrogen Deposition

**DOI:** 10.1371/journal.pone.0036257

**Published:** 2012-04-26

**Authors:** Wei-Ming He, Daniel Montesinos, Giles C. Thelen, Ragan M. Callaway

**Affiliations:** 1 State Key Laboratory of Vegetation and Environmental Change, Institute of Botany, the Chinese Academy of Sciences, Beijing, China; 2 Centro de Investigaciones sobre Desertificación-CIDE (CSIC-UV-GV), Apartado Oficial, Albal, Spain; 3 Division of Biological Sciences, the University of Montana, Missoula, Montana, United States of America; Jyväskylä University, Finland

## Abstract

Increased resource availability can promote invasion by exotic plants, raising concerns over the potential effects of global increases in the deposition of nitrogen (N). It is poorly understood why increased N favors exotics over natives. Fast growth may be a general trait of good invaders and these species may have exceptional abilities to increase growth rates in response to N deposition. Additionally, invaders commonly displace locals, and thus may have inherently greater competitive abilities. The mean growth response of *Centaurea stoebe* to two N levels was significantly greater than that of North American (NA) species. Growth responses to N did not vary among *C. stoebe* populations or NA species. Without supplemental N, NA species were better competitors than *C. stoebe*, and *C. stoebe* populations varied in competitive effects. The competitive effects of *C. stoebe* populations increased with N whereas the competitive effects of NA species decreased, eliminating the overall competitive advantage demonstrated by NA species in soil without N added. These results suggest that simulated N deposition may enhance *C. stoebe* invasion through increasing its growth and relative competitive advantage, and also indicate the possibility of local adaptation in competitive effects across the introduced range of an invader.

## Introduction

Soil nitrogen (N) is the most limiting factor for plant growth in most terrestrial ecosystems [1], [2] and human activity is increasing rates of N deposition [3]. High concentrations of N can promote dominance and invasion by exotic plants [4], [5]. The effects of increased N on exotic plants have been extensively studied [6]–[10], but several aspects of the effects of increased N on exotic plants remain poorly understood.

First, why should exotic invaders benefit more from higher N supply than natives? Good invaders tend to be fast growing species and fast growing species tend to be N-efficient, achieving relatively high carbon gain per unit of N [11]. Nitrate reductase activity, a key physiological mechanism in converting N into plant growth, has been reported to be higher in comparisons of groups of invasive species to native species [12]. Previous studies have shown that inter-specific competition shifts along a resource gradient [13]–[15]. Exotic invaders are also often good competitors, as indicated by very high relative abundances and community-scale decreases in native diversity and abundance [16], [17]–[22]. In addition to disproportional effects on growth, increased N may alter competition among native and exotic plant species. In grasslands, N-efficient species usually outcompete high N-requiring species [23], [24]; invaders, which appear to be strong competitors in general, tend to be high N-requiring species, often with higher leaf N concentrations than associated natives [25], [26]. Recent studies have shown that elevated soil N significantly affects the growth and competition of *Centaurea stoebe* [4], [5].

A second unexplored aspect of the effects of N deposition on invasion is variation among populations of invasive species. Genetic diversity within and among populations allows species to respond to selection imposed by competition [27]. For exotic invaders, substantial inter-population variation has been demonstrated for size, growth rate, herbivore defense, and morphological traits [28], but there is much less information on how populations of an invasive species may vary in competitive effects. Ridenour et al. (2008) found that NA populations of the European invader *C. stoebe* were affected less by competition than European populations, but they also found large differences in this response among populations within North America [28]. Reinhart and Rinella (2011) found that there were significant differences in competitive ability and allelopathic effects between eastern populations and western populations of *C. stoebe* in North America [18]. However, there have been no studies of inter-population variation in response to increased soil N.

*Centaurea stoebe* L. was introduced from Europe into North America in the late 1800s and is now among the most destructive invaders in North America [29]. *Centaurea stoebe* plants from NA populations have been shown to be larger than plants from European populations [30] and this species is a highly competitive invader [16], [17], [30]. *Centaurea stoebe* is now distributed throughout the United States and southern Canada, and these populations experience a wide range of N deposition rates with higher N deposition in northeastern regions and lower N deposition in northwestern regions. *Centaurea stoebe* tends to invade sandy, rocky and relatively nutrient poor soils (personal observation by Ray M. Callaway), raising the possibility that relatively small increases in N might cause large changes in plant communities invaded by *C. stoebe*.

In three different N conditions, we compared the growth of five populations of *C. stoebe* and their competitive effects on four NA species, and contrasted the growth and competitive effects of four NA species on *C. stoebe*. We addressed the following questions: (1) does N deposition increase growth and competitive advantages of *C. stoebe* more than those of native species? (2) do populations of *C. stoebe* show variation in growth and competition? (3) are the growth responses to N correlated with competitive effect?

## Materials and Methods

### Study species

Seeds of *C. stoebe* were collected from more than 10 individuals of one population in each of the following regions: Arkansas, Maryland, Montana and Vermont in the United States of America (USA), and from British Columbia in Canada. These collections, to some extent, represent a substantial portion of the range of *C. stoebe* in North America. We chose four NA common plants (i.e. *Helianthus annuus* L., *Vulpia octoflora* Rydb., *Achillea millefolium* L., and *Poa pratensis* L.) that represent a range of life histories and that are widely distributed in North America. These species also occur in each of the same general regions as all five *C. stoebe* populations. *Helianthus annuus* (common sunflower, Asteraceae) is an annual forb, *V. octoflora* (sixweeks fescue, Poaceae) is an annual grass, *A. millefolium* (common yarrow, Asteraceae) is a perennial forb, and *P. pratensis* (Kentucky bluegrass, Poaceae) is a perennial grass. The seeds of these four NA species were collected in the Missoula Valley, Montana.

### Experimental design

**Figure 1:**
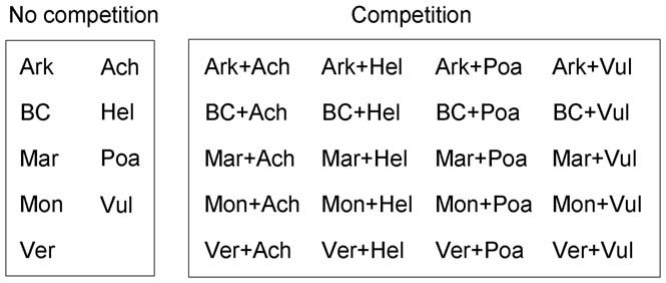
Experimental scheme. Ark = Arkansas, BC = British Columbia, Mar = Maryland, Mon = Montana, Ver = Vermont, Ach = *Achillea*, Hel = *Helianthus*, Poa = *Poa*, and Vul = *Vulpia*. Single abbreviations (the left panel; e.g., Ark or Ach) represent one plant grown alone (i.e. no competition), and two abbreviations together (the right panel; e.g., Ark+Ach) represent two plants grown together (i.e. competition). There were 14 and eight replicates for *Centaurea stoebe* populations (i.e. Ark, BC, Mar, Mon, and Ver) and North American species (i.e. Ach, Hel, Poa, and Vul) grown alone (i.e. no competition), respectively; there were seven replicates for the pair-wise competition.

We conducted a greenhouse experiment at The University of Montana, and thus no specific permits were required for this study. Plants from the five populations of *C. stoebe* were each planted in competition with each of the four NA species (i.e. two plants per pot, one *C. stoebe* and one competitor), testing inter-specific competition (Figure 1; the right panel). Plants from all *C. stoebe* populations and all NA species were also grown alone as controls (Figure 1; the left panel, no competition). There were 14 and eight replicates for *C. stoebe* populations and NA species grown alone, respectively. For each species-population pair we had seven replicates. Since our goal was to contrast the growth and competitive effects of both *C. stoebe* and NA species at different potential N deposition regimes we repeated this entire design three times, once for “ambient” N concentrations, which consisted of Montana field soils that are a sandy, rocky and nutrient poor soil (i.e. 0.99 mg g^−1^ and 7.54 mg g^−1^ for NH_4_^+^ and NO_3_^−^), again for an estimation of low deposition rates (1 g N m^−2^ yr^−1^), and a third time for an estimation of high deposition rates (4 g N m^−2^ yr^−1^). According to the National Atmospheric Deposition Program/National Trends Network (NADP/NTN, see http://nadp.sws.uiuc.edu), current N deposition ranges between 0.1 and 2.0 g N m^−2^ yr^−1^ across the United States. Thus we chose 1 g N m^−2^ yr^−1^ as a low deposition rate. Based on the previous experiments and predictions for future deposition rates [31]–[33], we used 4 g N m^−2^ yr^−1^ as a high deposition rate. These applications represent reasonable experimental treatments for atmospheric deposition, but these levels are quite low relative to N that can be added through other management or natural processes [34].

Of the related studies we were able to find in the literature, 55% used ammonium nitrate (NH_4_NO_3_) in experiments simulating N deposition; thus we added N as NH_4_NO_3_ in solution in de-ionized water in our experiment. Specifically, the total amounts of 32.2 mg and 128.8 mg NH_4_NO_3_ were added to each pot for the low and high deposition rates on three dates (i.e. May 5, June 5, and July 5). During the course of the experiment, all plants were watered every 2–3 days depending on how fast the soil dried. All plants were grown from seeds and grown in 535 cm^3^ (upper diameter: 12 cm; lower diameter: 6 cm; height: 25 cm) pots filled with local soil from Missoula, Montana, which is a nutrient poor soil. All pots were randomly arranged on one bench and rotated per week. Greenhouse temperatures were maintained between 15–30°C, corresponding roughly with natural summer temperatures. Natural light in the greenhouse was supplemented by metal halide bulbs, and total photosynthetically active radiation during the day remained above 1200 µmol m^−2^ s^−1^. The experiment lasted for four months (from planting seeds to harvesting plants), from March 28, 2009 to July 27, 2009, and at the end of the experiment, all plants were harvested, washed, dried at 60°C for three days, and then the whole-plant biomass was determined.

### Data analyses

To quantify the effects of N deposition on the growth of *C. stoebe* populations and NA species, the increased growth was calculated as: (*B_n_*−*B_c_*)/*B_c_*×100%, where *B_n_* is the biomass of a plant grown alone and subjected to N addition (i.e. low N deposition and high N deposition) and *B_c_* is the mean biomass of all the plants grown alone but subjected to no N addition. To quantify the effects of N deposition on competition intensity, we calculated relative interaction intensity (RII) through the following method: RII = (*B_w_*−*B_o_*)/(*B_w_*+*B_o_*), where *B_w_* is the biomass of a plant when growing with a neighboring plant and *B_o_* is the mean biomass of all the plants growing alone [35]. RII has values ranging from −1 to 1, and is negative for competition and positive for facilitation [35]. In our study, we modified the above equation so that *B_w_* was the biomass of a plant of each of the five *C. stoebe* populations or each of the four NA species, in competition with a neighbor, and *B_o_* was the mean biomass of all the plants of each of the five *C. stoebe* populations or each of the four NA species, grown alone under a given N regime. RII was calculated for each N level (i.e. no N addition, low N addition, and high N addition) separately. For each species-population pair, each individual had its own RII value to be used in ANOVA. When three N levels were considered together, the values of RII were pooled. For more details, see the figure legends for each case.

For five *C. stoebe* populations, we used a two-way ANOVA to test the effects of N deposition, population origin, and their interaction on the increased growth and RII of *C. stoebe* populations. By the same token, for four NA species, we also used a two-way ANOVA to test the effects of N deposition, species identity, and their interaction on increased growth and RII of four NA species. Additionally, a two-way ANOVA was also used to test the effects of N and species identity (i.e. invasive versus local species) when all *C. stoebe* populations and NA species were considered together. To explore the competitive effects among *C. stoebe* populations across three N regimes, we used a one-way ANOVA to test the effects of population origin on each of the four NA species or on all four NA species together. Correlation analysis was used to determine the relationships between RII and increased growth. If there was a correlation between RII and increased growth, then we regressed mean RII of all *C. stoebe* populations and for each NA species against mean growth responses to N for all *C. stoebe* populations and for each NA species. All statistical analyses were conducted using SPSS 13.0.

## Results

### Nitrogen addition versus growth and competitive effects

**Figure 2:**
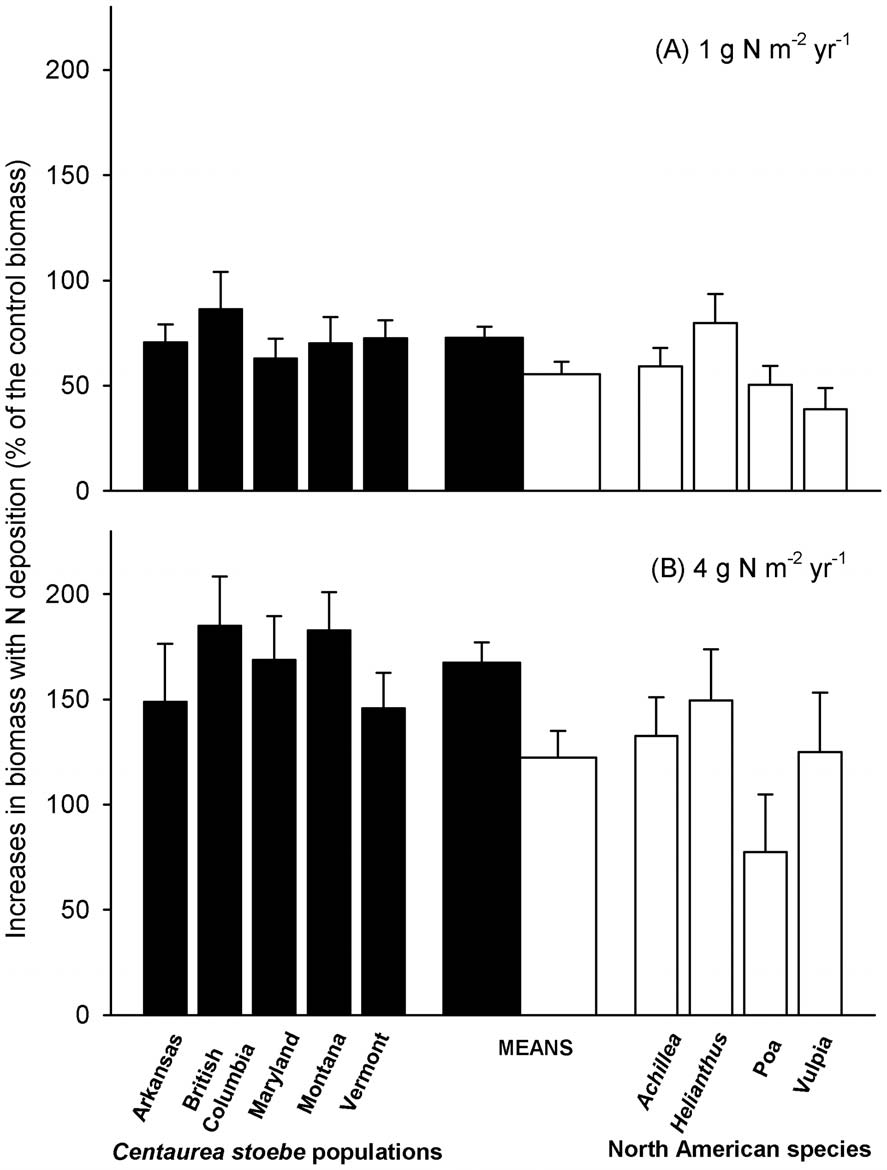
Effects of (A) low and (B) high nitrogen addition on biomass (means+1 SE) of five *Centaurea stoebe* populations (narrow black bars) and four North American (NA) species (narrow white bars). Wide bars are means (+1 SE) for all five *C. stoebe* populations (black) or all four NA species (white) combined. Increases in biomass = (*B_n_*−*B_c_*)/*B_c_*×100%, where *B_n_* was the biomass of a plant of each of the five *C. stoebe* populations or each of the four NA species, grown alone and subjected to low or high N deposition; *B_c_* was the mean biomass of all the plants of each of the five *C. stoebe* populations or each of the four NA species, grown alone but subjected to no N addition.

Nitrogen addition increased the mean growth of *C. stoebe* across all populations from 72% at low N deposition rates to 168% with high N deposition rates (*F* = 199.9, *df* = 1,124, *P*<0.001), but there were no significant differences among *C. stoebe* populations (*F* = 2.44, *df* = 4,121, *P* = 0.204) (Fig. 2). Nitrogen addition also enhanced the mean growth of NA species, from 56% under the low N deposition to 122% under the high N deposition (*F* = 25.51, *df* = 1,63, *P* = 0.013); there were no differences in this response among NA species (*F* = 4.19, *df* = 3,61, *P* = 0.135) (Fig. 2). When all *C. stoebe* populations and NA species were considered together, higher N concentrations increased the growth of *C. stoebe* more than that of NA species. At low N deposition concentrations the increased growth was 72±5% versus 56±6% (Fig. 2A; *F* = 4.01, *df* = 1,88, *P* = 0.048), and at high N deposition the increased growth was 168±9% versus 122±13% (Fig. 2B; *F* = 7.98, *df* = 1,99, *P* = 0.006).

**Figure 3:**
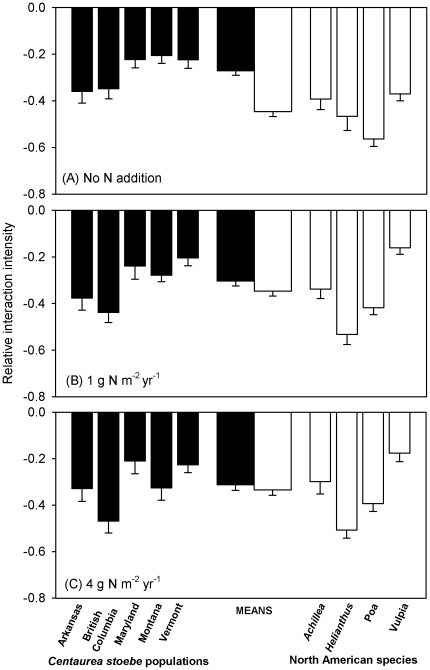
The mean competitive effects (means+1 SE), as indicated by relative interaction intensity (RII = (*B_w_*−*B_o_*)/(*B_w_*+*B_o_*)), of five *Centaurea stoebe* populations on four North American (NA) species (narrow black bars) and of four NA species on five *C. stoebe* populations (narrow white bars) under (A) no N addition, (B) low and (C) high N addition. Wide bars show means (+1 SE) for all five *C. stoebe* populations (black) or all four NA species (white). RII was calculated for each population under a given N level across four NA species, and *B_w_* and *B_o_* were the biomass of a plant of each of the four NA species in competition with a *C. stoebe* plant and the mean biomass of all the plants of each of the four NA species grown alone. RII was calculated for each NA species under a given N level across five *C. stoebe* populations, and *B_w_* and *B_o_* were the biomass of a plant of each of the five *C. stoebe* populations in competition with a NA plant and the mean biomass of all the plants of each of the five *C. stoebe* populations grown alone.

Relative interaction intensity (RII) differed substantially among *C. stoebe* populations (*F* = 15.25, *df* = 4,315, *P* = 0.001) and among NA species (*F* = 12.29, *df* = 3,316, *P* = 0.006) when three N regimes were considered together (Fig. 3). The mean RII for five *C. stoebe* populations on NA species differed among species (*F* = 29.85, *df* = 3,316, *P*<0.001) and among N levels (*F* = 13.01, *df* = 2,317, *P* = 0.001) (Fig. 3). There was a N by species interaction for RII of *C. stoebe* on NA species (*F* = 2.45, *df* = 6,308, *P* = 0.025), indicating that N affected the response of some species to competition with *C. stoebe* but not others. This interaction between N and annuals was stronger than that between N and perennials. We did not find N by population interactions for the response of *C. stoebe* to NA species (*F* = 0.79, *df* = 8,305, *P* = 0.616). Across all *C. stoebe* populations and all NA species, the competitive effect of NA species was significantly greater than that of *C. stoebe* when no N addition was supplied (Fig. 3A; −0.271±0.019 for *C. stoebe* versus −0.447±0.021 for NA species; *F* = 38.92, *df* = 1,119, *P*<0.0001); there were no differences in RII at low N supply (Fig. 3B; −0.304±0.021 for *C. stoebe* versus −0.347±0.021 for NA species; *F* = 2.12, *df* = 1,97, *P* = 0.146) or high N supply (Fig. 3C; −0.313±0.024 for *C. stoebe* versus −0.335±0.023 for NA species; *F* = 0.46, *df* = 1,101, *P* = 0.497). In other words, NA species lost this competitive advantage when N was increased.

### Variation in growth and competitive effects among populations

**Figure 4:**
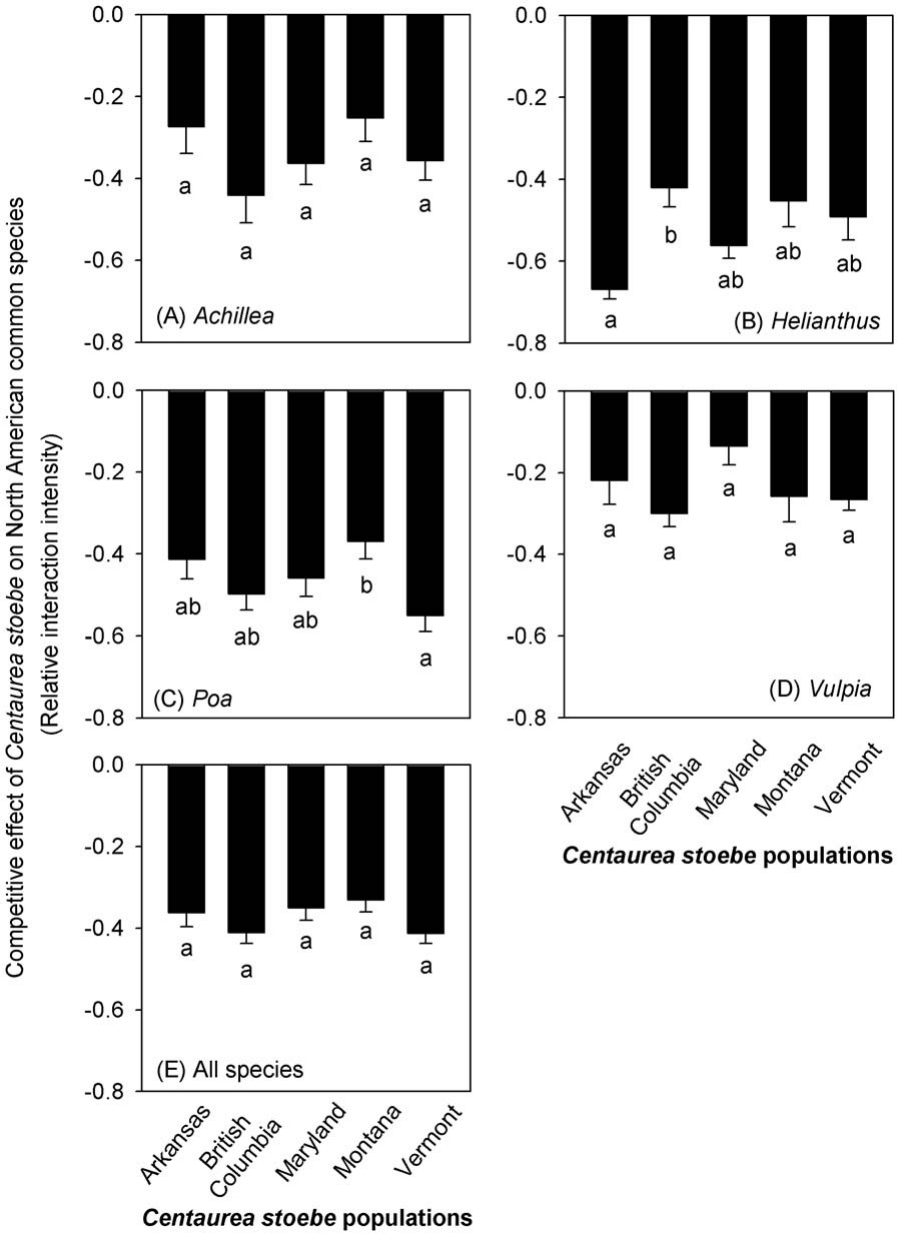
The competitive effects, as indicated by relative interaction intensity (RII = (*B_w_*−*B_o_*)/(*B_w_*+*B_o_*)), of different *C. stoebe* populations on the four North American (NA) species across three nitrogen levels. Bars show means+1 SE for each *C. stoebe* population. RII was calculated for each population in competition with a given NA species across three N levels, and *B_w_* and *B_o_* were the biomass of a plant of a given NA species in competition with a *C. stoebe* plant and the mean biomass of all the plants of a given NA species grown alone.

The response of *C. stoebe* to competition varied among populations, we also found substantial differences in the competitive effects of *C. stoebe* populations on *P. pratensis* and *H. annuus* (Fig. 4). However, the competitive effects of different *C. stoebe* populations were not consistent among NA species. For example, plants from the *C. stoebe* population in Arkansas suppressed *H. annuus* more than plants from the British Columbia population. However, plants from the Arkansas population were not the strongest competitors against *P. pratensis*. *Centaurea stoebe* plants from Vermont were better competitors against *P. pratensis* than plants from the Montana population, but they were not the best competitors against any other NA species.

### Relationship between growth and competitive effects

**Figure 5:**
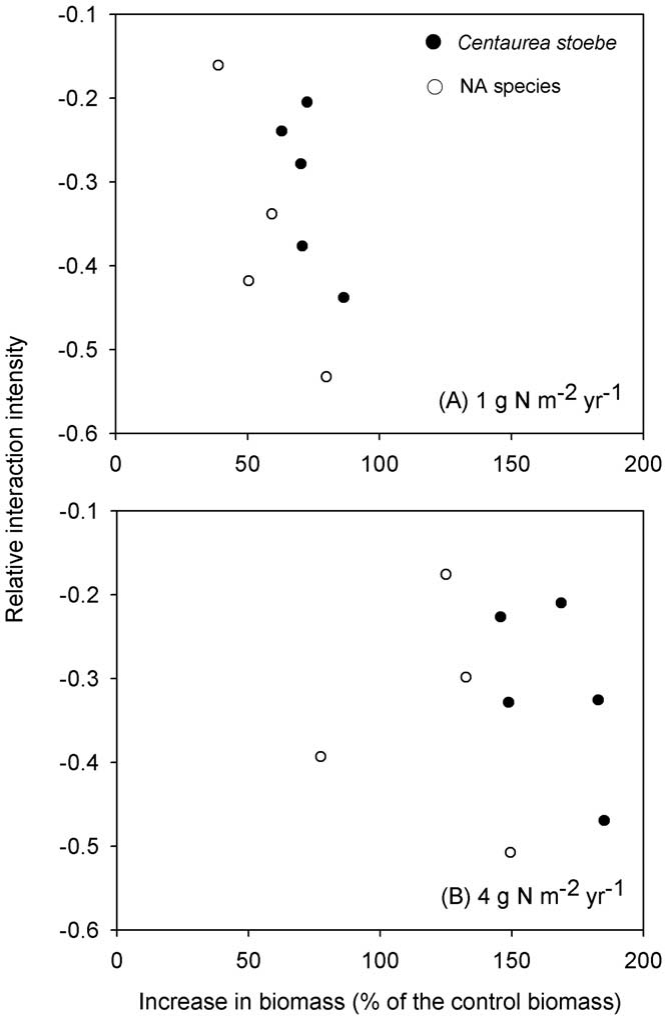
Correlation between relative interaction intensity of *Centaurea stoebe* populations and North American (NA) species and increases in biomass of *C. stoebe* populations and NA species when grown alone under the low N addition (A) and high N addition (B). The points in Figure 5 correspond to the values of increased growth (Figure 2) and RII (Figure 3B & C). For more details on the calculations of increased growth and RII, see above.

Mean RII values of *C. stoebe* populations across four NA species were negatively correlated with the mean increase in biomass of *C. stoebe* plants from ambient N concentrations to low N deposition concentrations (*r* = −0.723, *P* = 0.084), so were the mean RII values of NA species negatively correlated with their growth response (*r* = −0.877, *P* = 0.061) (Fig. 5A). The regression equations were: RII = −0.008×increased growth+0.287 (*R* ^2^ = 0.522) and RII = −0.007×increased growth+0.089 (*R* ^2^ = 0.769) for the *C. stoebe* populations and NA species, respectively. By contrast, at high N there were no significant correlations between RII and increased biomass when plants were subjected to high N deposition and grown alone, regardless of *C. stoebe* populations or NA species (all *P*>0.05; Fig. 5B), suggesting that competitive effect was independent of increased growth in higher N deposition.

## Discussion

### Nitrogen deposition increases the growth and competitive advantages of invaders

Our results provide evidence that N deposition differentially affects the growth and competitive advantages of *C. stoebe* and NA species, suggesting that the overall growth and competitive advantages of invaders may be enhanced by N deposition. Our results are consistent with previous findings that inter-specific competition shifts along a resource gradient [13]–[15]. Additionally, our findings also add to an understanding of the potential consequences of N deposition for the risks of plant invasions.

Nitrogen added to soil to mimic anthropogenic deposition rates enhanced the growth of the exotic perennial herb *C. stoebe* more than that of NA species, but higher N eliminated the strong competitive advantages that NA species had in soils without added N. These effects of N on growth correspond with many studies in the literature [31], [36], [37], and also consistent with our results for *C. stoebe* many others have found that high N levels confer competitive advantages of invaders over common species [4], [5], [10], [38]–[41]. Thomsen et al. (2006) conducted an experiment with three common perennial grasses (*Agrostis oregonensis*, *Festuca rubra*, and *Nassella pulchra*) and three exotic perennial grasses (*Holcus lanatus*, *Phalaris aquatica*, and *Festuca arundinacea*), and found that elevated soil N did not influence competition among the common and exotic perennial grasses [24]. Abraham et al. (2009) contrasted the performances of exotic and common plant species with annual and perennial life histories with increased N availability and found species-specific results [42]. The perennial *Nassella pulchra* did not respond to N addition when grown alone, and competed best against the exotic annual grass *Bromus diandrus* at low N. In their experiment two common perennial grasses were suppressed more by *B. diandrus* than the exotic perennial grass, *Holcus lanatus*, which had the greatest response of all the perennials to N additions when grown alone. Knochel et al. (2010) found that *C. stoebe* exhibited a large biomass response to N addition, but the presence of grasses suppressed the ability to exploit this N [4]. Our recent research suggests that N deposition enhances the growth of the annual invader *Bromus tectorum* and maintains its inherent competitive advantages [10]. Our findings, in conjunction with previous studies, demonstrate that community outcomes could be determined by which species are present at the time management is applied, the specific levels of N under consideration, or limitation by other resources. High N supply seems to tip competitive outcomes in favor of invaders, and thus increases the relative abundance of exotic species.

### Spatial patterns of growth and competitive effects of *C. stoebe* populations

We did not find significant differences in the growth potential among *C. stoebe* populations, inconsistent with previous findings that elevated soil N significantly affects the growth of *Centaurea stoebe* [4], [5]. The variation in competitive effect we measured among *C. stoebe* populations suggests a high level of species-specificity in the way *C. stoebe* interacts with other species [20], and the potential for substantial local adaptation across the non-native range of the invader. Similarly, Fridley et al. (2007) found that when different intraspecific genotypes were grown without neighbors the growth of the genotypes did not differ among environmental treatments [43]. But when grown in species mixtures, the performance of different genotypes was significantly different depending on the neighboring species. In high fertility treatments, the performance of different genotypes did not alter competitive outcomes. At low fertility, competitive dominance was affected by genetic identity. Crutsinger et al. (2007) found that high diversities of different genotypes of *Solidago altissima* reduced the biomass of colonizing species more than low diversities of genotypes [44], suggesting that different genotypes can elicit different competitive effects. A more recent study shows that there are significant differences in competitive ability and allelopathic effects between eastern populations and western populations of *C. stoebe* in North America [18]. In addition, the growth and competitive advantages of invaders in response to N addition may vary with native and introduced ranges [10]. Our results suggest that inter-population variation for invaders exists for competitive interactions, as previous experiments have shown for size, growth rate, herbivore defense, and morphological traits [28].

### Correlations between competitive effects and enhanced growth

In our study, mean competitive effects across four NA species were negatively correlated with the mean increased size of plants from different populations of *C. stoebe* at low N supply, so were such patterns for NA species at low N supply. In other words, across the small range of plant size in our experiments, bigger plants were better competitors. This is consistent with our recent experiment with an annual invader *B. tectorum* [10]. Across a larger range of *C. stoebe* plant size, individual plant biomass or growth rate must certainly affect competitive interactions, but our results may have been due to allelopathic effects of *C. stoebe* [16], [20], [45] or to trade-offs between growth and other traits [21]. Weir et al. (2010) pointed out that the role of root exudates may be limited [46]. A recent study has shown that the eastern populations of *C. stoebe* in USA may have no allelopathic effects and the opposite is the case for western populations [18]. In contrast, the mean competitive effect of NA commons was positively correlated with the mean size of the species at the low N supply. There were no correlations between competitive effect and size at the high N supply, regardless of *C. stoebe* or NA plant species. Thus this correlation may vary with annual and perennial invaders.
